# Evolution of pneumococcal serotype epidemiology in Botswana following introduction of 13-valent pneumococcal conjugate vaccine

**DOI:** 10.1371/journal.pone.0262225

**Published:** 2022-01-05

**Authors:** Sweta M. Patel, Yazdani B. Shaik-Dasthagirisaheb, Morgan Congdon, Rebecca R. Young, Mohamed Z. Patel, Tiny Mazhani, Sefelani Boiditswe, Tirayaone Leburu, Kwana Lechiile, Tonya Arscott-Mills, Andrew P. Steenhoff, Kristen A. Feemster, Samir S. Shah, Coleen K. Cunningham, Stephen I. Pelton, Matthew S. Kelly

**Affiliations:** 1 Division of Pulmonary, Allergy and Critical Care Medicine, Duke University, Durham, NC, United States of America; 2 Duke Global Health Institute, Duke University, Durham, NC, United States of America; 3 Division of Pediatric Infectious Diseases, Boston University School of Medicine, Boston, MA, United States of America; 4 Division of General Pediatrics, Children’s Hospital of Philadelphia, Philadelphia, PA, United States of America; 5 Division of Pediatric Infectious Diseases, Duke University, Durham, NC, United States of America; 6 Department of Paediatric and Adolescent Health, Faculty of Medicine, University of Botswana, Gaborone, Botswana; 7 Botswana—University of Pennsylvania Partnership, Gaborone, Botswana; 8 Division of Pediatric Infectious Diseases and Global Health Center, Department of Pediatrics, Children’s Hospital of Philadelphia, Philadelphia, PA, United States of America; 9 Divisions of Hospital Medicine and Infectious Diseases, Cincinnati Children’s Medical Center, Cincinnati, OH, United States of America; 10 Department of Pediatrics, University of California, Irvine, Irvine, CA, United States of America; Universidade de Lisboa Faculdade de Medicina, PORTUGAL

## Abstract

Pneumococcal conjugate vaccines reduce the burden of invasive pneumococcal disease, but the sustained effect of these vaccines can be diminished by an increase in disease caused by non-vaccine serotypes. To describe pneumococcal serotype epidemiology in Botswana following introduction of 13-valent pneumococcal conjugate vaccine (PCV-13) in July 2012, we performed molecular serotyping of 268 pneumococcal strains isolated from 221 children between 2012 and 2017. The median (interquartile range) age of the children included in this analysis was 6 (3,12) months. Fifty-nine percent of the children had received at least one dose of PCV-13 and 35% were fully vaccinated with PCV-13. While colonization by vaccine serotypes steadily declined following PCV-13 introduction, 25% of strains isolated more than 3 years after vaccine introduction were PCV-13 serotypes. We also observed an increase in colonization by non-vaccine serotypes 21 and 23B, which have been associated with invasive pneumococcal disease and antibiotic resistance in other settings.

## Introduction

*Streptococcus pneumoniae* is a leading cause of child mortality worldwide. While pneumococcal conjugate vaccines effectively prevent invasive pneumococcal disease (IPD) caused by vaccine serotypes, their use has been accompanied by an increase in disease caused by non-vaccine serotypes [1]. Changes in nasopharyngeal carriage of pneumococcal serotypes often parallel changes in serotypes that cause IPD [2]. Carriage studies can thus provide valuable information on the impact of pneumococcal vaccines in countries lacking robust IPD surveillance. We sought to describe temporal trends in pneumococcal serotype carriage among children in Botswana following introduction of 13-valent pneumococcal conjugate vaccine (PCV-13).

## Methods

### Study setting and design

Botswana introduced PCV-13 (Prevnar-13; Pfizer) into its immunization program in July 2012. The vaccine is administered at 2, 3, and 4 months of age and was introduced without a catch-up campaign. The estimated national coverage rate for the complete vaccine series in infants ranged from 52% after PCV-13 introduction in 2012 to 89% in 2017 [3]. This research used nasopharyngeal swab samples collected from children under 24 months of age enrolled in two prospective cohort studies in Botswana between April 2012 and December 2017. Samples collected between 2012 and 2016 were obtained from children enrolled in a prospective cohort study of infants and young children with and without pneumonia, the design of which was previously described in detail [4]. Briefly, children 1–23 months of age who either presented to a tertiary hospital in Gaborone with symptoms meeting the World Health Organization (WHO) definition of pneumonia or who presented for routine or acute care at a public clinic in the Gaborone area were eligible for enrollment. Exclusion criteria included the presence of a chronic medical condition predisposing to pneumonia (other than HIV infection), hospitalization in the prior 14 days, and asthma. One nasopharyngeal swab was collected from each child at the time of enrollment. Samples collected between 2016 and 2017 were obtained from infants enrolled in a birth cohort study of mother-infant pairs recruited within 72 hours of delivery in and around Gaborone, Botswana. Exclusion criteria for this study included birth weight <2000 g, birth by Caesarian section, and multiple gestation pregnancy. Infants were seen every 1 to 2 months until they reached 12 months of age, and a nasopharyngeal swab was collected at each visit. In both studies, children of mothers with documented negative HIV testing at or prior to enrollment were classified as HIV-unexposed, uninfected. Children of mothers with HIV were classified as HIV-exposed, uninfected (HEU) if they tested negative for HIV at or after 6 weeks of age if exclusively formula fed, at least 6 weeks after breastfeeding cessation, or at enrollment.

### Laboratory methods

Nucleic acid from nasopharyngeal samples was extracted on the NucliSens easyMAG platform and tested for *S*. *pneumoniae* using a quantitative PCR assay targeting the autolysin gene (*lytA*) gene [5]. Samples with >500 copies/mL of *lytA* were considered to have detectable *S*. *pneumoniae* DNA and underwent real-time sequential multiplex PCR as previously described [6] with primers characterized at Centers for Disease Control and Prevention (CDC) that detect 40 common serotypes or serogroups [7]. Real-Time PCR (RT-PCR) reagents (SsoAdvanced Universal Supermixes) were purchased from Bio-Rad Laboratories (Hercules, CA). PCR primers and probes were synthesized by Sigma-Millipore (St. Louis, MO). RT-PCR conditions used were as follows: 95°C for 15 min, followed by 35 amplification cycles of 94°C for 30 sec, 54°C for 90 sec, and 72°C for 60 sec. A final hold was performed at 72°C for 10 min. RT-PCR assays were performed according to manufacturer instructions using a CFX96 Touch Real-Time PCR Detection System (Bio-Rad, Hercules, CA). Before the multiplex assays, primers were tested using serotype-specific positive and negative controls for specificity. A Ct value ≤35 was considered positive for the reaction. In addition to the Ct value, melting curves of each reaction were carefully evaluated before considering results for the reaction. Cultures were not performed. For children with more than one nasopharyngeal sample collected over time, the earliest sample from which *S*. *pneumoniae* was detected was chosen for serotyping.

### Statistical analysis

We classified serotypes/serogroups into PCV-13 serotypes (1, 3, 4, 5, 6A, 6B, 7F/A, 9V/A, 14, 18C/F/B/A, 19A, 19F, 23F) and non-vaccine serotypes (2, 6C/D, 11A, 12F, 15A/F, 15B, 16F, 21, 22F, 23A, 23B, 33F, and 35B). Analyses were limited to nasopharyngeal samples with identifiable pneumococcal serotypes by PCR. We compared categorical variables using Chi-square or Fisher’s exact tests and continuous variables using Wilcoxon rank sum tests. We used multinomial logistic regression, adjusted *a priori* for age and HIV exposure status, to evaluate for an association between collection year and serotype category [8–11]. Statistical analyses were conducted using R version 3.6.2.

### Ethical statement

Written informed consent was obtained from parents or legal guardians for all children enrolled in this study. This study was approved by the Health Research and Development Committee (Ministry of Health, Botswana) and institutional review boards at Princess Marina Hospital, the University of Pennsylvania, McMaster University, Boston University, and Duke University.

## Results

### Patient characteristics

**Fig 1:**
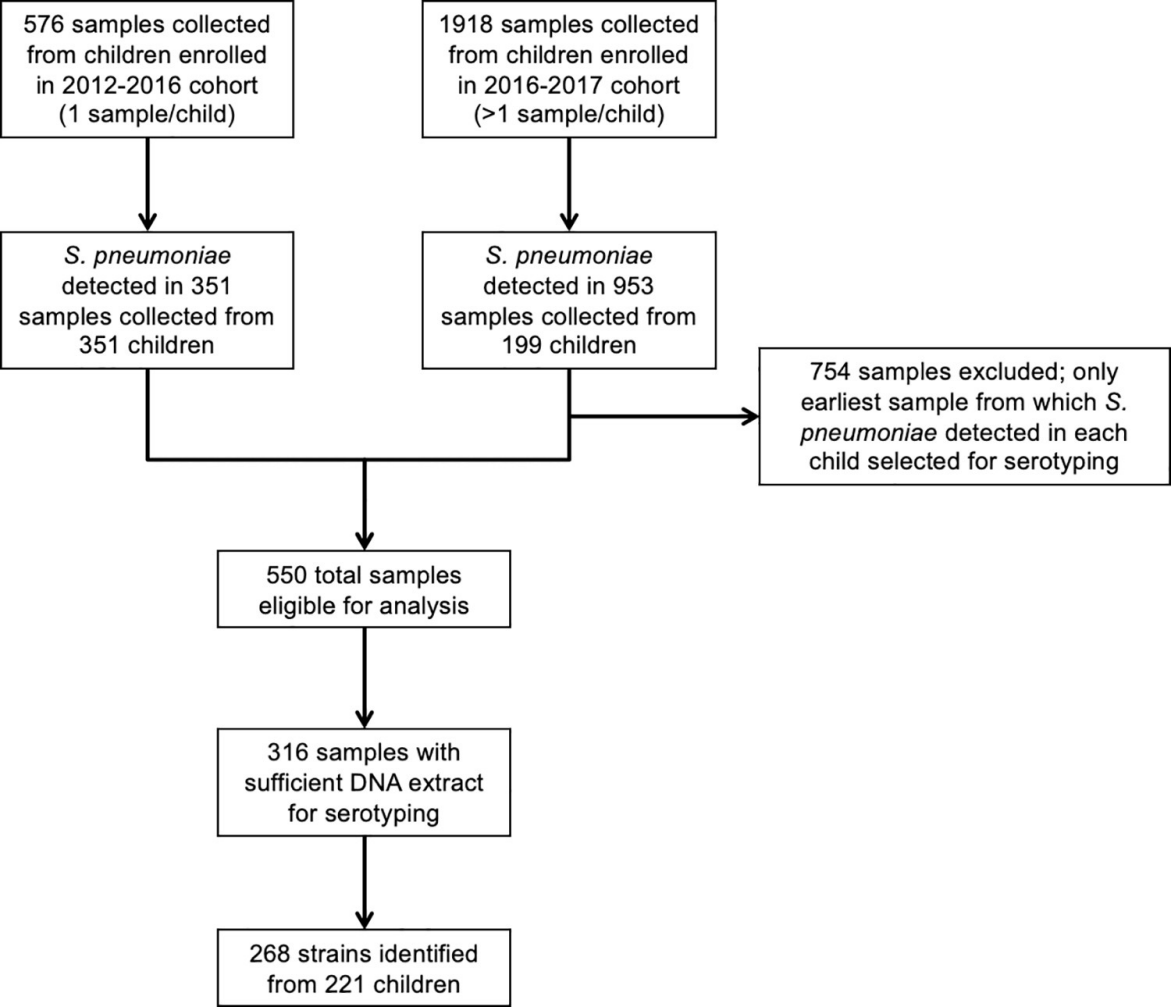
Specimen collection and selection for pneumococcal serotyping.

*S*. *pneumoniae* was identified in 1,304 of 2,494 (52%) nasopharyngeal samples collected from 550 of 841 (65%) enrolled children. Sufficient DNA extract was available for serotyping samples from 316 children, and we identified 268 *S*. *pneumoniae* strains from a final study population of 221 children (**Fig 1**). Of the 221 children included in this analysis, 124 (56%) children had pneumonia. Thirty-eight (17%) children were concurrently colonized with two or more serotypes. The number of nonserotypeable samples identified by year during the study period is shown in **S1 Table**. Characteristics of the study population are shown in **Table 1**. Median (IQR) age of children with PCV-13 serotypes was higher than that of children with non-vaccine serotypes [8 (3, 14) months vs. 4 (2, 10) months; *P* = 0.001], but we did not observe a difference in serotype classification by HIV status, sex, or number of PCV-13 doses received. Forty-eight percent of strains isolated from fully vaccinated children were PCV-13 serotypes.

**Table 1:** Characteristics of the study population (n = 221).

Characteristic		Total N (%)	2012–2016 cohort (N = 160)	2016–2017 cohort (N = 61)	*P*
Age in months (median, IQR)		6 (3, 12)	9 (4, 15)	2 (1, 4)	**<0.001**
Female sex		110 (50)	73 (46)	37 (61)	0.05
HIV exposure status^a^ (n = 213)					**0.01**
	HIV-infected	9 (4)	9 (6)	0 (0)	
	HIV-exposed, uninfected	63 (30)	38 (25)	25 (41)	
	HIV-unexposed	141 (66)	105 (69)	36 (59)	
PCV-13 doses received (n = 220)					**0.01**
	0	91 (41)	59 (37)	32 (53)	
	1	29 (13)	18 (11)	11 (18)	
	2	23 (11)	17 (11)	6 (10)	
	3	77 (35)	66 (41)	11 (18)	

Abbreviations: IQR, interquartile range.

^a^ Children were classified as HIV-unexposed, uninfected if their mothers had documented negative HIV testing at or prior to enrollment; children whose mothers tested positive for HIV before or at delivery were classified as HIV-exposed, uninfected if they tested negative for HIV after 6 weeks of age if exclusively formula fed, at least 6 weeks after breastfeeding cessation, or at enrollment.

### Serotype epidemiology over time

**Fig 2:**
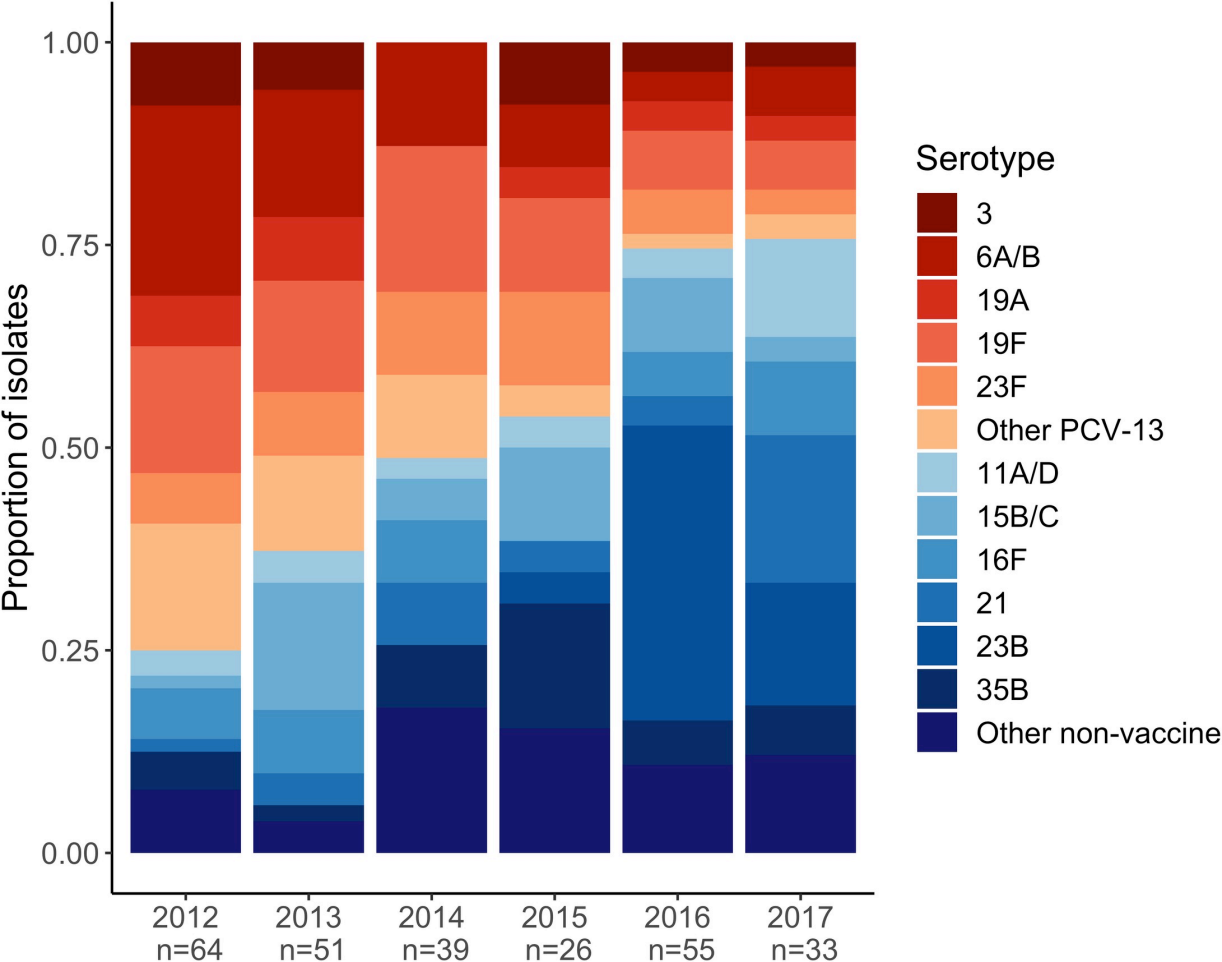
Serotypes of pneumococcal strains isolated from infants and young children in Botswana. The bars represent the serotypes of identified pneumococcal isolates collected from children in Botswana by year of enrollment. Vaccine serotypes are shaded red to orange, while non-vaccine serotypes are presented in shades of blue. The number of colonizing isolates serotyped is displayed below the enrollment year.

The serotypes isolated over the course of the study period are shown in **Table 2**. The proportion of PCV-13 serotypes declined from 80 of 115 (70%) isolates in 2012–2013 to 22 of 88 (25%) isolates in 2016–2017 (**Fig 2**). The most common serotypes isolated in 2012–2013 were 6A/6B and 19F. In 2016–2017, the most frequently identified serotypes were 21 and 23B. Four percent of strains between 2012–2015 were classified as serotype 21, compared to 9% of strains in 2016–2017 (P = 0.09). One percent of strains between 2012–2015 were classified as serotype 23B, compared to 28% of strains in 2016–2017 (P < 0.001). The likelihood of a pneumococcal strain being a PCV-13 serotype decreased by 36% per year during the study period (relative risk 0.64; 95% confidence interval: 0.54, 0.76, *P*<0.001). The detailed results of the multinomial regression analysis are shown in **Table 3**. Among children enrolled between 2012 and 2016, the proportion of strains that were classified as PCV-13 was similar in children with pneumonia compared to healthy children (60% vs 67%, respectively; *P* = 0.43).

**Table 2:** Serotypes by vaccine classification and year.

Serotypes	2012	2013	2014	2015	2016	2017	Totals
**PCV-13**	**48**	**32**	**20**	**12**	**14**	**8**	**134**
1	2	0	1	0	0	0	3
3	5	3	0	2	2	1	13
4	0	2	1	0	0	0	3
5	1	0	0	0	0	0	1
6 A/B	15	8	5	2	2	2	34
9V/A	5	3	0	0	0	1	9
14	2	0	2	1	0	0	5
18C/F/B/A	0	1	0	0	1	0	2
19A	4	4	0	1	2	1	12
19F	10	7	7	3	4	2	33
23F	4	4	4	3	3	1	19
**Non-PCV-13**	**16**	**19**	**19**	**14**	**41**	**25**	**134**
6C/D	0	0	0	0	1	0	1
11A/D	2	2	1	1	2	4	12
12F/A/B/44/46	0	0	1	2	1	1	5
15A/F	1	1	1	0	3	2	8
15B/C	1	8	2	3	5	1	20
16F	4	4	3	0	3	3	17
21	1	2	3	1	2	6	15
22F/A	2	0	4	1	0	0	7
23A	2	1	1	1	1	0	6
23B	0	0	0	1	20	5	26
33F/33A/37	0	0	0	0	0	1	1
35B	3	1	3	4	3	2	16
**Total #**	**64**	**51**	**39**	**26**	**55**	**33**	**268**

**Table 3:** Full results of multinomial regression analysis.

Variable	Estimated regression coefficient	Standard error of regression coefficient	Exponentiated coefficient	Exponentiated 95% Confidence Intervals (CI)	P value
Time elapsed since start of study	-0.449	0.087	0.638	0.538, 0.756	<0.001
Age (months)	0.009	0.023	1.009	0.964, 1.056	0.70
HIV exposure without infection	0.181	0.751	1.198	0.275, 5.223	0.81
No HIV exposure	0.216	0.719	1.241	0.303, 5.086	0.76

## Discussion

PCV-13 introduction was associated with a substantial reduction in nasopharyngeal carriage of vaccine serotypes among children in Botswana. Despite high national vaccine uptake, however, the proportion of colonizing strains that were PCV-13 serotypes remained high (25%) five years after vaccine introduction. Our findings are consistent with prior studies in children conducted after PCV-13 introduction in South Africa, where vaccine serotypes comprised 22% of colonizing pneumococcal isolates, and Malawi, where vaccine serotypes were isolated from 23% of children [12, 13]. However, our results contrast with prior studies conducted in England and Belgium, where the prevalence of carriage of vaccine serotypes declined to <6% within five years of PCV-13 introduction [14, 15]. These discordant findings could reflect differences in herd immunity due to varied vaccine administration schedules or population prevalence of HIV infection or other immunocompromising conditions. Additionally, the pneumococcal antibody thresholds needed to protect against nasopharyngeal carriage among children in low- and middle-income countries may be higher than the thresholds needed in high-income countries [16]. As PCV-13 serotypes remain responsible for a large proportion of IPD cases in low- and middle-income countries despite increasing use of PCV-13, residual carriage in these settings may blunt the impact of pneumococcal vaccination on childhood mortality [17].

We also observed changes in the prevalence of several pneumococcal serotypes commonly implicated in clinical disease. Early in the study period, serotypes 6A, 6B, and 19F were the most commonly identified serotypes. Strains belonging to serogroups 6 and 19 were among the most common causes of IPD prior to the introduction of conjugate vaccines, and reduced circulation of these serotypes may have contributed to the decline in hospitalizations and deaths due to childhood pneumonia in Botswana following PCV-13 introduction [17, 18]. By comparison, the serotype epidemiology in Botswana following PCV-13 introduction differs from other settings in sub-Saharan Africa. For example, serotype 15B/C became the dominant pneumococcal serotype in South Africa after PCV-13 introduction [12], but the proportion of serotype 15B/C isolates remained relatively stable over time among children in our cohort. During the final two years of our study, the most common serotypes identified were the non-vaccine serotypes 21 and 23B. Though it is unclear if this increase in serotype 23B will be sustained, its rise is particularly concerning given that it has become one of the most common serotypes isolated from children with IPD following PCV-13 introduction in other settings and is often resistant to common antimicrobials [15, 19]. The current contribution of these serotypes to IPD in Botswana is unknown, but their emergence illustrates the need for more effective vaccines to prevent pneumococcal infections during childhood.

This study has a number of limitations. First, the study population was composed of children from two distinct cohorts that differed based on characteristics including age and PCV-13 vaccination status. For example, the association between age and pneumococcal serotype epidemiology may be confounded by time period, because children enrolled between 2012 and 2016 were older than children enrolled between 2016 and 2017. Additionally, the inclusion of children with pneumonia may have biased our results towards the identification of PCV-13 serotypes. However, the proportion of children with pneumonia colonized with PCV-13 serotypes was similar to that of healthy controls enrolled during the same study period. Furthermore, children were recruited from a geographically restricted area of Botswana, which limits the generalizability of our findings. The molecular assays that we used identified only 40 of the over 100 known pneumococcal serotypes. We also did not identify a serotype in approximately one-third of samples that underwent molecular serotyping, which likely reflects the presence of serotypes not identified by the CDC primers, non-typeable strains, or other *Streptococcus* species containing the *lytA* gene. While CDC primers encompass all PCV-13 serotypes, many non-vaccine serotypes are not represented in the CDC primer set; consequently, pneumococcal strains that were present in the nonserotypeable samples were more likely to be non-vaccine serotype strains and our decision to exclude these samples from our analysis may have biased our results. Finally, we only had data on colonizing serotypes from young children and were unable to evaluate for changes in serotype epidemiology among the strains that cause IPD or colonize adults.

In summary, following PCV-13 introduction in Botswana, we observed a reduction in nasopharyngeal carriage of vaccine serotypes accompanied by an increase in carriage of non-vaccine serotypes associated with IPD and antibiotic resistance in other settings. While existing vaccines dramatically reduced pneumococcal disease among children worldwide, further gains will depend on strategies to strengthen herd immunity and address the rise of clinically significant non-vaccine serotypes through the development of expanded valency or non-capsule-based pneumococcal vaccines.

## Supporting information

S1 Table Classification of all lytA+ samples. (DOCX)
