# Supplementary material for: Evolution of pneumococcal serotype epidemiology in Botswana following introduction of 13-valent pneumococcal conjugate vaccine
Source: PLoS One. 2022 Jan 5;17(1):e0262225. doi: 10.1371/journal.pone.0262225 (PMC8730465; doi:10.1371/journal.pone.0262225)
Supplement: S1 Table — (DOCX) [file pone.0262225.s001.docx]

**Evolution of pneumococcal serotype epidemiology in Botswana following introduction of 13-valent pneumococcal conjugate vaccine**

**Supplemental File**

| **Table S1.** Classification of all *lytA+* samples | | | | | | | |
| --- | --- | --- | --- | --- | --- | --- | --- |
| **Serotypes** | **2012** | **2013** | **2014** | **2015** | **2016** | **2017** | **Totals** |
| Samples containing only PCV-13 strains | 36 | 24 | 17 | 12 | 8 | 6 | 103 |
| Samples containing only non-PCV-13 strains | 11 | 14 | 15 | 13 | 27 | 20 | 100 |
| Samples containing both PCV-13 and non-PCV-13 strains | 4 | 4 | 2 | 0 | 6 | 2 | 18 |
| *lytA+* samples without identified serotype | 15 | 16 | 21 | 21 | 4 | 18 | 95 |
| **Total #** | **66** | **58** | **55** | **46** | **45** | **46** | **316** |
